# An unusual case of recurrent haemoptysis after ablation for atrial fibrillation requiring pneumonectomy: a case report

**DOI:** 10.1093/ehjcr/ytae140

**Published:** 2024-03-15

**Authors:** Giovanni Mattioni, Riccardo Orlandi, Barbara Rubino, Andrea Garatti, Ugo Pastorino

**Affiliations:** Thoracic Surgery Unit, IRCCS National Cancer Institute of Milan, Via Giacomo Venezian, 1, 20133 Milano, MI, Italy; School of Thoracic Surgery, University of Milan, Via Festa del Perdono, 7, 20122 Milano, MI, Italy; Thoracic Surgery Unit, IRCCS National Cancer Institute of Milan, Via Giacomo Venezian, 1, 20133 Milano, MI, Italy; School of Thoracic Surgery, University of Milan, Via Festa del Perdono, 7, 20122 Milano, MI, Italy; Pathology and Cytology Unit, IRCCS Galeazzi-Sant’Ambrogio Hospital, Via Cristina Belgioioso, 173, 20157 Milano, MI, Italy; Cardiac Surgery Unit, IRCCS Policlinico San Donato, Piazza Edmondo Malan, 2, 20097 San Donato Milanese, MI, Italy; Thoracic Surgery Unit, IRCCS National Cancer Institute of Milan, Via Giacomo Venezian, 1, 20133 Milano, MI, Italy

**Keywords:** Pulmonary vein stenosis, Iatrogenic, Atrial fibrillation, Catheter ablation, Haemoptysis, Pneumonectomy, Case report

## Abstract

**Background:**

Pulmonary vein (PV) stenosis is a rare complication after catheter ablation for atrial fibrillation (AF). While there have been reported anecdotal cases of complete PV stenosis requiring pulmonary lobectomy, only one case of pneumonectomy has been documented so far.

**Case summary:**

A 42-year-old man was referred to our Thoracic Surgery Unit for recurrent haemoptysis and exertional dyspnoea over the past 4 years and a recent finding of left PV occlusion. He suffered of relapsing AF that had almost five recurrences and that underwent a total of two percutaneous catheter ablations within a 7-year period. He also experienced a hospitalization for multifocal lobar pneumonia. Two attempts of percutaneous transluminal angioplasty (PTA) were unsuccessful. Due to the severity and the duration of PV occlusion, the previous PTA failure, the patient’s age, and his symptoms, a left pneumonectomy was performed. During the postoperative period, the patient experienced only mild anaemia effectively managed with blood transfusions. Five months after surgery, he has no recurrence of symptoms.

**Discussion:**

When the PV stenosis is complete, PTA may face high failure and recurrence rates. In this setting, anatomical pulmonary resections may represent a valid option to allow symptom relief and resolution.

**Learning points**

- The occurrence of haemoptysis and/or respiratory infections following a percutaneous catheter ablation procedure for atrial fibrillation should raise the suspicion of iatrogenic pulmonary vein stenosis.
- When facing a complete and longstanding pulmonary vein stenosis, anatomical lung resection can be considered as a resolutive treatment.

## Introduction

Pulmonary vein (PV) stenosis is an uncommon complication after percutaneous catheter ablation for atrial fibrillation (AF). The stenosis usually involves a single PV on one side.^1^ The severity of the stenosis may vary from mild to complete. When clinically relevant, it may occur with exertional dyspnoea, cough, fatigue, chest pain/discomfort, haemoptysis, and recurrent respiratory infections. Few cases requiring pulmonary lobectomy have been described in literature; however, only one pneumonectomy has been reported so far.^2^ Here, we describe the case of a patient suffering from iatrogenic left PV complete stenosis. This article has been written following the SCARE guidelines.^3^

## Summary figure

**Figure:**
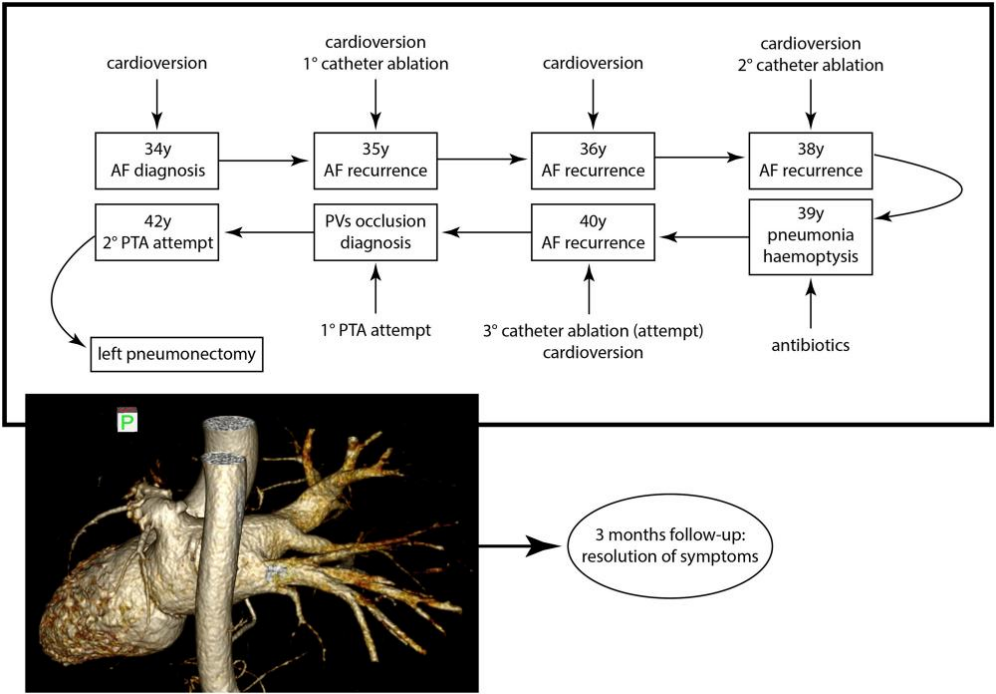


## Case presentation

**Figure 1:**
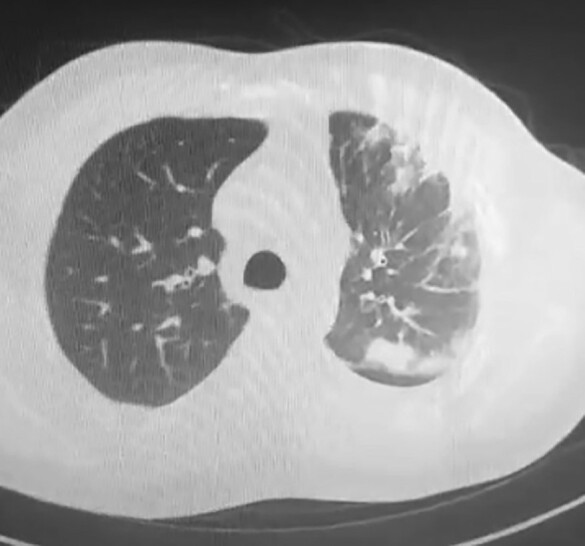
Computed tomography scan of the thorax showing multiple lung opacities at the left upper lobe, along with ipsilateral pleural effusion.

**Figure 2:**
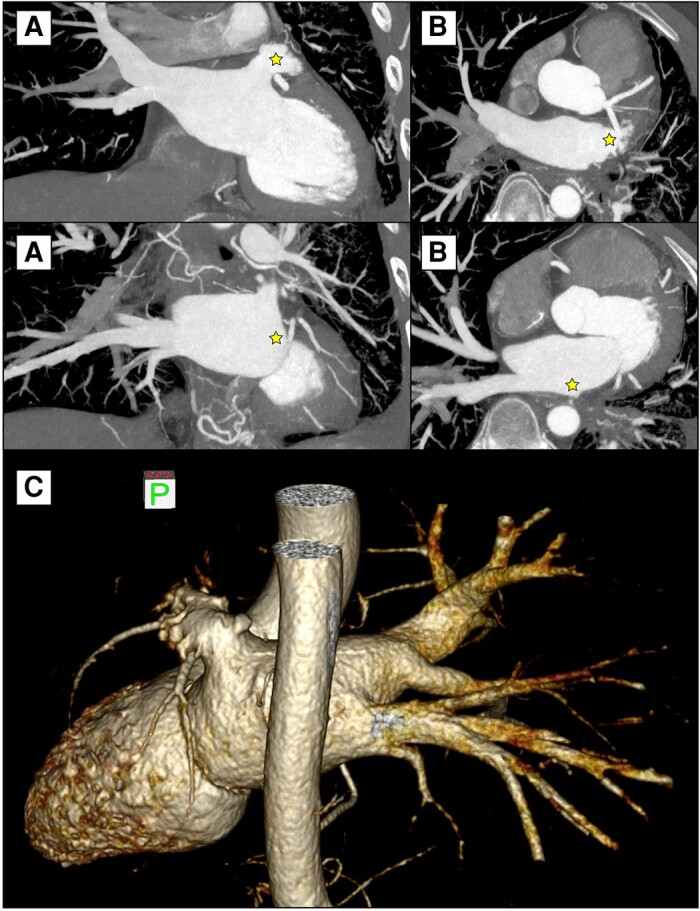
Computed tomography scans of the coronal (*A*) and transverse (*B*) sections showing interruption of the left upper pulmonary vein and absence of the left inferior pulmonary vein (star). (*C*) Posterior view of the 3D reconstruction of the left atrium, left ventricle, and aorta.

**Figure 3:**
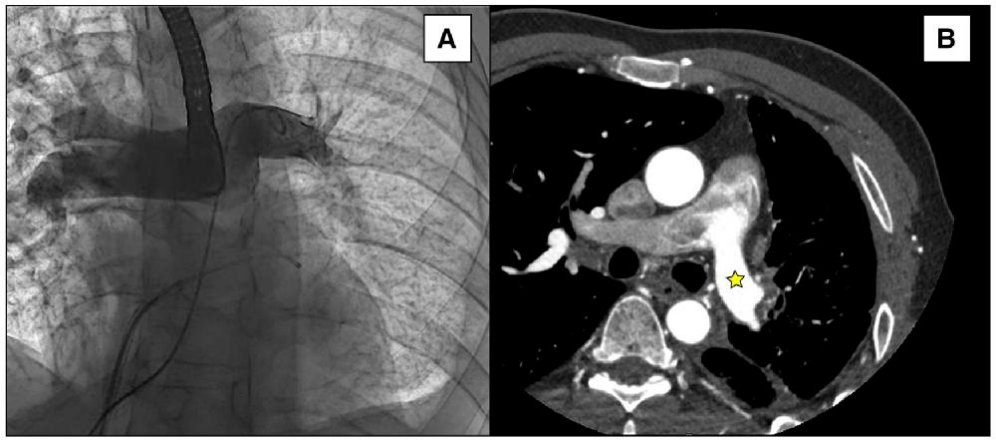
(*A*) Pulmonary artery angiography (under transoesophageal ultrasonography guidance) showing absence of contrast agent flow in the distal part of the left pulmonary artery. (*B*) Computed tomography scan showing stagnation of contrast agent in the left pulmonary artery (star).

A 42-year-old Asian man was referred to our Thoracic Surgery Unit for recurrent haemoptysis and exertional dyspnoea over the past 4 years and a recent finding of left PV occlusion. He suffered from relapsing AF, which was first diagnosed at the age of 34 as a symptomatic tachycardia-related cardiomyopathy. He had a recent diagnosis of type 2 insulin-dependent diabetes, had a history of smoking (18 pack-years), and worked as a cook. He was on medications, including apixaban, bisoprolol, low-dose digoxin, and amiodarone. At AF diagnosis, in 2015, he received electrical cardioversion. He then experienced AF recurrence in 2016, 2017, 2019, and 2021, requiring cardioversion (both electrical and pharmacological). A catheter cryoablation was performed in 2016 (details unavailable), followed by another ablation in 2019 using radiofrequency. During the latter one, PV mapping indicated right PV disconnection and electrical conductance in the left PVs. Entering the mapping catheter in the left PVs was reported to be technically challenging due to anatomical reasons. The procedure concluded without complications and with a successful electrical cardioversion. In 2020, he was hospitalized for dyspnoea, fever, and haemoptysis. The chest computed tomography (CT) scan showed multifocal pneumonia of the left upper lobe and pleural effusion (*Figure 1*); a stenosis of the left upper PV was not recognized due to the absence of contrast agent. At bronchoscopy, marked hyperaemia of the left upper lobe bronchus was found. He received antibiotics and was discharged after clinical improvement. In 2021, due to AF recurrence, a third catheter ablation was initiated but abandoned due to the presence of haemoptysis. The patient therefore underwent a contrast-enhanced CT scan showing complete stenosis of both left PVs (*Figure 2*). A percutaneous transluminal angioplasty (PTA) was attempted but given the impossibility of passing the guidewire through the left PVs and the complete absence of venous flow at transoesophageal echocardiography, the procedure was aborted. In 2023, he underwent another PTA attempt, which was again unsuccessful. During this procedure, the left pulmonary artery was catheterized, confirming an absence of contrast enhancement of the distal third of the artery (*Figure 3*; Supplementary material online, *Video S1*). The patient was then referred to our centre for surgical evaluation.

**Figure 4:**
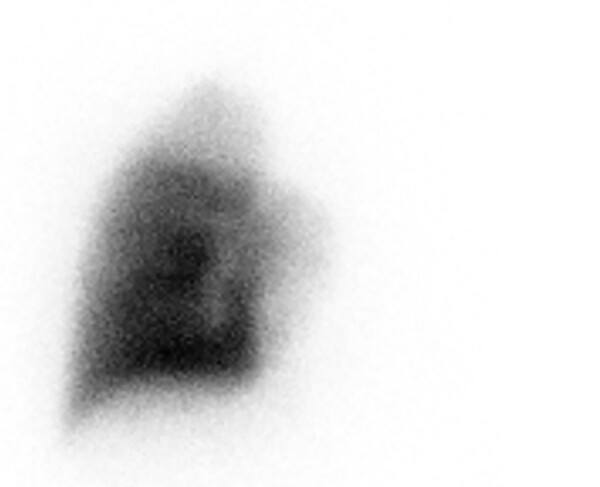
Lung scintigraphy showing absence of perfusion to the left lung.

The electrocardiogram showed sinus rhythm, and echocardiography showed an ejection fraction (EF) of 64% with an estimated pulmonary artery pressure of 56 mmHg. Lung perfusion scan showed absence of perfusion of the left lung (*Figure 4*). Respiratory function test reported a forced expiratory volume in one second of 82%, a forced vital capacity of 90%, a diffusing capacity of the lungs for carbon monoxide (DLCO) of 67%, and a DLCO/alveolar volume of 100%, and the blood oxygen saturation was 98% at rest at room air. The CT scan displayed a significant hypoplasia of the left lung, a completely closed left inferior PV, and a truncated upper left PV (at 1.6 cm from the atrial origin, *Figure 2*). In this condition, the left lung represented only a source of bleeding and infection and did not contribute to gas exchange. Given the severity and the duration of PV occlusion and the previous PTA failure, the surgical option was the only available. The patient’s age and his symptoms favoured an invasive treatment. A restoration of the vein patency was believed to be highly unlikely. After multidisciplinary discussion, a left pneumonectomy was proposed.

Digoxin and apixaban were discontinued before the operation.

## Surgical procedure

The patient was placed in a semi-supine position, and a left hemi-clamshell incision was made, accessing the pleural cavity through the fifth intercostal space. The lung surface appeared rich of vascular reticulation and congested. Diffuse, dense, vascularized pleuropulmonary adhesions were found and lysed. Furthermore, numerous hypertrophic bronchial arteries were identified at the lung hilum and ligated. The left upper PV appeared hypoplastic, while the inferior vein was unidentifiable within dense fibrous tissue. Macroscopical examination of the surgical specimen revealed diffused hepatization (see Supplementary material online, *Figure S1*). The postoperative course was characterized by mild anaemia treated with blood transfusions. The chest tube was removed at postoperative day (POD) 2, and the patient was discharged at POD 7. At 5 months of follow-up, he had no relapse of AF and haemoptysis, and the exertional dyspnoea improved. The final pathological report showed pleural sclerosis, diffuse alveolar hyperplasia, interalveolar septa fibrosis, and vascular congestion, along with intra-alveolar haemorrhage (see Supplementary material online, *Figure S2*).

## Discussion

Iatrogenic PV stenosis following AF catheter ablation is relatively rare, with an estimated incidence of 1–3%.^1^ Complete stenosis (i.e. occlusion) is even rarer, with an incidence ranging 0.8–1.3%^4^ of procedures. Symptoms may widely vary and could occur even months after the ablation, as exertional dyspnoea, cough, fatigue, chest pain/discomfort, haemoptysis, and recurrent respiratory infections. Imaging may show multifocal lobar pneumonia, lung consolidation, or intra-alveolar haemorrhage. Although bronchoscopy may not reveal abnormalities, marked hyperaemia can occasionally be found.^5,6^ The stenosis can affect any PV, but the right superior, the left superior, and the left inferior PVs are more commonly involved (30% incidence each). The anatomy of the stenosis is also variable and can involve the origin of the vein or a more distal segment.^1^ Pulmonary artery flow reduction or cessation is a potential complication of vein occlusion,^4^ as reported in our case. Clinically relevant PV stenosis may be treated with PTA. However, when complete (i.e. occlusion), it may occasionally require an anatomical lung resection, due to a low probability of restoring sufficient patency through PTA.^1^ The catheter passage is often unsuccessful, and restenosis risk is high. In addition, severe complications may occur, as venous rupture and haemorrhage.^7^

Only 10 cases of lung resection have been reported in literature (Supplementary material online, *Table S1*) and of them, one was a pneumonectomy.^2,5,6,8–14^ In one additional case, resection was aborted due to severe pleuropulmonary adhesions.^11^ Mean age at surgery was 47.7 years (median 50.5, range 17–71). A video-assisted thoracic surgery approach was described in three cases. Intraoperatively, diffuse pleuropulmonary adhesions were usually encountered, and congestion of the involved lobe with hypertrophic superficial vessels were also found. No postoperative complications were recorded. On the contrary, Fender *et al.*^15^ described two additional cases undergoing lung resection following restenosis after PTA that experienced postoperative death due to bleeding. However, no further details are available. When follow-up was reported, the mean duration was 12 months (median 6, range 3 weeks–33 months), and, noteworthy, no recurrence of symptoms was noted. Actually, an additional case of pneumonectomy can be found in literature; however, it was performed in a more complex setting: a patient suffering from left PV stenosis that underwent PTA complicated by venous rupture, resulting in haemorrhage and cardiac tamponade.^7^ Rare cases of PV reconstruction have been described; however, a case series for stenosis >70% showed a 38% recurrence rate during long follow-up.^16^ In the context of both stenosis and occlusion of different veins, a combination of lung resection and vein reconstruction has also been reported.^10^

To the best of our knowledge, this is the second case of pneumonectomy for complete stenosis of unilateral PVs following AF catheter ablation. Notably, the main difference from the first case is that our patient presented haemoptysis and respiratory infection, received more than one ablation procedure, and had a longstanding PV occlusion.^2^ Our experience confirmed that surgery is successful in definitively relieving life-threating symptoms. However, this procedure should be performed exclusively in high-volume centres after multidisciplinary evaluation with proper cardiothoracic expertise and in carefully selected patients. Given the rarity of this condition, each case deserves individual assessment. As a rule, anatomical lung resection should be taken into account in case of fit patients, only after previous failed vein PTA or restenosis, or with longstanding PV stenosis and severe symptoms.

## Conclusion

Iatrogenic complete PV stenosis is a rare complication of catheter ablation of AF. Lung resection can occasionally represent a valid therapeutic option in this setting. When occlusion involves both PVs on one side, pneumonectomy may achieve satisfactory results in carefully selected cases.

## Supplementary Material

ytae140_Supplementary_Data

## Data Availability

The data underlying this article will be shared upon reasonable request to the corresponding author.

## Lead author biography

I am a thoracic surgery resident in Milan, Italy, and I obtained my medical degree in Perugia. Now, I am in Paris, France, for a training period. The field of thoracic surgical oncology is my major interest.

## Supplementary material

**Consent:** Consent for publication has been obtained, in line with the COPE best practice guidelines, and that the individual who is being reported on is aware of the possible consequences of that reporting.

**Funding:** None declared.
