# Supplementary material for: An unusual case of recurrent haemoptysis after ablation for atrial fibrillation requiring pneumonectomy: a case report
Source: Eur Heart J Case Rep. 2024 Mar 15;8(4):ytae140. doi: 10.1093/ehjcr/ytae140 (PMC10990059; doi:10.1093/ehjcr/ytae140)
Supplement: ytae140_Supplementary_Data [file ytae140_supplementary_data.zip › Supplementary.docx]

**Supplementary materials and legends**

Supplementary Table S1. List of studies reporting lung resections for PVs complete stenosis.

| Study | Age | Sex | Symptoms | N. of ablation procedures | Involved PV | Treatment | Complications | Follow-up |
| --- | --- | --- | --- | --- | --- | --- | --- | --- |
| Ravenel J.G. et al. 2002 | 31y | M | Haemoptysis, chest pain, CT- scan left upper lobe GGO | 1 | LSV | Left upper lobectomy | None | Not reported |
| Venkataraman R. et al. 2023 | 71y | F | Dyspnoea, left pleural effusion | 1 | LSV, LIV | Open left pneumonectomy | None | 2 months |
| Papakonstantinou N. et al. 2018 | 50y | M | Left upper lobe pneumonia, haemoptysis, CT-scan lung infiltrates | 2 | LSV | Open left upper lobectomy | Not reported | Not reported |
| Cheng S. et al. 2018 | 37y | M | Massive haemoptysis, CT-scan GGO at Left upper lobe | 2 | LUV | VATS left upper lobectomy | Not reported | 6 months |
| Janilionis R. et al. 2017 | 54y | M | Chest pain, cough, haemoptysis | 1 | LLV | Left lower lobectomy | Not reported | Not reported |
| Lo CM. et al. 2016 | 47y | F | Right lower lobe pneumonia, haemoptysis chest discomfort | 1 | RLV | VATS right lower lobectomy | None | 18 months |
| Murray L. et al. 2022 | 68y | M | CT-scan left lower lobe consolidation, cough, dyspnoea | 1 | LLV (+ partial stenosis LUL) | 1° VATS left lower lobectomy + 2° open (sternotomy) left upper vein angioplasty | Not reported | Not reported |
| O’Gorman K. et al. 2019 | 51y | M | Haemoptysis, cough, exertional dyspnoea, fever, CT-scan multifocal infiltrates at left upper lobe. | 1 | LUV | Aborted left upper lobectomy (previous PTA) | NA | NA |
| Steliga M. et al. 2010 | 51y | F | Cough chest pain, CT-scan GGO at left upper lobe | 1 | LUV | Open left upper lobectomy | None | 3 weeks |
| Libretti L. et al. 2012 | 17y | M | Recurrent pneumonia | Not reported | LLV | Left lower lobectomy (previous PTA) | Not reported | 33 months |

Supplementary Video S1. Pulmonary artery angiography during transoesophageal echocardiography monitoring, showing interruption of contrast flow in the left pulmonary artery.


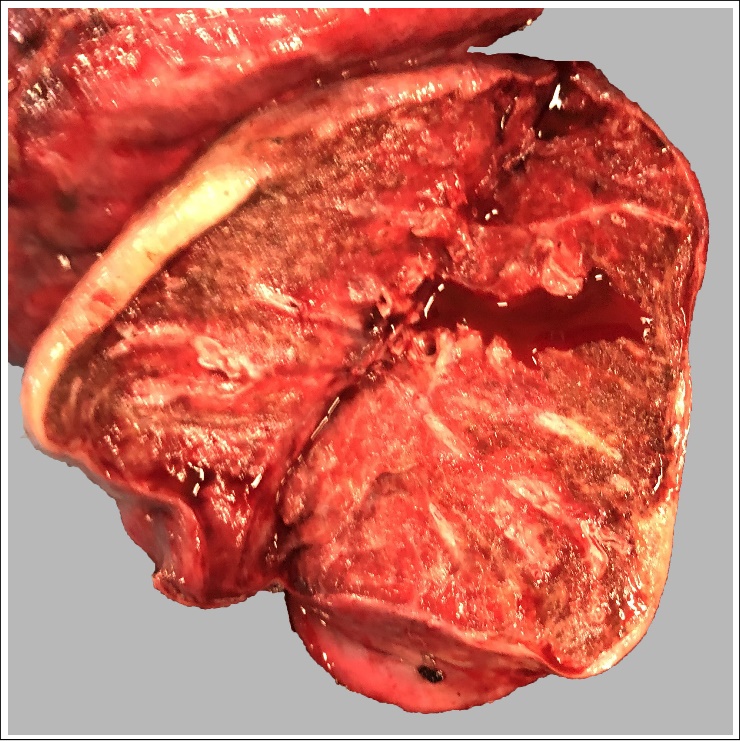


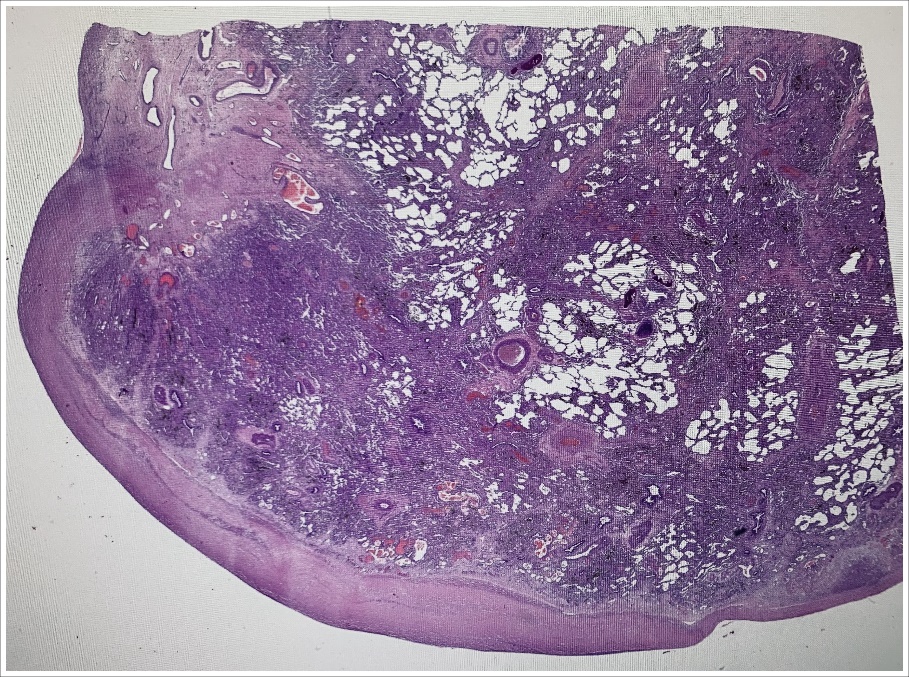
Supplementary Figure S1. Surgical specimen of the left lower lobe incised and opened.

Supplementary Figure S2. Pathological image of the lung, showing fibrous thickening of pleura and interlobular septa, along with intimal hyperplasia and medial thickening of both pulmonary veins and arteries (EE 2x).
